# Validation of HCL-33 in screening for bipolar disorder in patients with depressive episode

**DOI:** 10.1192/j.eurpsy.2023.829

**Published:** 2023-07-19

**Authors:** K. Kola, F. Elezi

**Affiliations:** 1 Psychiatric Hospital Elbasan, Elbasan; 2 Neuroscience, University Health Clinic, Tirane, Albania

## Abstract

**Introduction:**

Hypomania Check List 33 (HCL-33) constitutes a self-rating questionnaire for lifetime history of hypomanic symptoms, used in patients who present with depressive symptoms and a further screening for bipolar disorder is required.

**Objectives:**

- Translation and cultural adaptation of HCL-33

- Measuring psychometric abilities, gender and age differences in score

**Methods:**

In order to culturally adapt, standardize and validate the instrument for the Albanian context, the reliability and validity of the HCL-33 was examined using an sample of 99 patients, of whom 22 were diagnosed as bipolar disorder but all presented with a depressive episode.

In order to reach reliability, internal consistency analyses were performed.

**Results:**

The factor analysis yielded two factor, with an internal consistency of .838 and .736 from the Cronbach’s alphas, with a total alpha of .765, falling within the “good to excellent” range.

Furthermore, Albanian norm scores and cut-off scores have been generated for the Albanian version of HCL-33. The article provides evidence regarding the psychometric propërties and utility of HCL-33 in the Albanian adult population for clinical assessment, outcome evaluations and research purposes. With a cut-off value of 16, sensitivity was 73% and specificity was 53%, with a prevalence of 22% and positivie predictive value of 30% and negative predictive value 87%.

Gender differences were not relevant in the total scoring, but there was a positive correlation between age an HCL-33 scoring (r(n=99)=.243, p≤.01), especially with Factor II (r(n=99)=.211, p≤.05).

**Conclusions:**

The article provides evidence regarding the psychometric properties and utility of HCL-33 in the Albanian adult population as a screening tool for bipolar disorder in patients presenting with a depressive episode.

**Disclosure of Interest:**

None Declared

